# A rare and massive retroperitoneal leiomyoma: a case report

**DOI:** 10.3389/fonc.2026.1842018

**Published:** 2026-06-01

**Authors:** Sujuan Jiang, Tingting Bai, Zhongwen Zou, Mengying Wang, Wenjing Xu, Mengze Xu, Kaiting Huang, Jie Wang, Mingwei Lv, Xue Li, Xiaoguang Shao, Jie Zhang

**Affiliations:** 1 Department of Obstetrics and Gynecology and Maternal-Child Reproductive Genetics Center, Affiliated Zhongshan Hospital of Dalian University, Dalian, Liaoning, China; 2 Department of Obstetrics and Gynecology, Dalian University, Dalian, Liaoning, China

**Keywords:** massive retroperitoneal leiomyoma, MRI, PET-CT, psychological intervention, surgery

## Abstract

**Objective:**

The objective of this case report is to describe the challenging surgical procedure of a rare, massive retroperitoneal leiomyoma and administration of postoperative circulation reconstruction, while addressing the patient’s significant perioperative psychological distress.

**Case presentation:**

A 52-year-old woman presented with fever and oliguria for 1 week. She presented with a giant pelvic and abdominal mass measuring 48×48×25 cm^3^. After multidisciplinary preparation and rectification of the mentioned detrimental symptoms, she underwent transabdominal resection with total hysterectomy and bilateral salpingo-oophorectomy. To prevent hemodynamic instability during tumor removal, a warm water bag was applied *in situ* during slow lifting of the mass. The 40-kg massive retroperitoneal neoplasm resulted in that the right kidney and ureter were displaced toward the abdominal midline. Throughout the tumor resection procedure, the upper one-third segment of the ureter was inadvertently transected, and a ureteral stent was placed. Postoperative pathological examination confirmed a spindle cell tumor in the right adnexal region, accompanied by hyaline degeneration and infarction, consistent with degenerative changes of leiomyoma. To reduce the risk of postoperative volume redistribution, we carried out rigorous management intraoperatively and postoperatively. Meanwhile, the patient’s Hamilton Anxiety Scale (HAMA) score decreased from 21 preoperatively to 9 postoperatively, with a reduction rate exceeding 50%, confirming a marked psychological therapeutic effect.

**Discussion and conclusions:**

This case emphasizes the critical role of multidisciplinary collaboration in the management of complex retroperitoneal masses and suggests that active clinical intervention can still yield favorable outcomes for patients with untreated conditions. It is worth emphasizing that psychological intervention is of great significance during her initial consultation, and empathetic and encouraging methods might be able to help her face this illness and receive treatment earlier.

## Introduction

Retroperitoneal leiomyomas are rare, with only a few case reports. Among them, the largest one to date was reported by Shi Y, measuring 40×30×25cm^3^ (1). Massive retroperitoneal masses represent a rare and challenging clinical issue in gynecological practice, with diverse origins, involving both gynecological and non-gynecological diseases. These masses can be benign or malignant, with significant variations in symptoms and management approaches (2).

Extrauterine leiomyomas located in retroperitoneum are rare, which may lead to misdiagnosis. While imaging can provide detailed information about the neoplasm and nearby neurovascular landmarks, histopathology remains the gold standard for definitive diagnosis. Appropriate surgical techniques are crucial for improving effects and recovery (3). Histologically, the distinction between benign leiomyoma and malignant leiomyosarcoma is difficult. The most difficult diagnostic problem is the distinction between a benign smooth muscle tumor and a low-grade leiomyosarcoma. Histological parameters used include gross tumor size, the presence of necrosis, cellularity, and nuclear pleomorphism (4).

Primary retroperitoneal masses, defined as tumors arising from the mesenchymal, neurogenic, or lymphatic tissues native to this space (rather than adjacent organs) (5), are exceedingly rare and are often malignant (3). Benign primary retroperitoneal tumors are exceptionally rare, with smooth muscle tumors being a notable subset (6).

Primary pelvic retroperitoneal smooth muscle tumors are very rare, with unknown etiology, and are often discovered incidentally. The retroperitoneal space is large and loose, which allows tumors to grow covertly, often without symptoms in the early stages. Symptoms only appear once the tumor has grown considerably, manifesting as abdominal distension, pain, and urinary frequency. These symptoms are related to signs caused by compression of adjacent structures (7).

The present article reports a rare case of the largest primary retroperitoneal leiomyoma. This report uniquely describes an innovative warm−water bag compression technique for intraoperative hemodynamic stabilization and details a standardized perioperative psychological assessment and treatment.

## Case report

A 52-year-old postmenopausal woman presented with a 10-year history of a progressively enlarging abdominal mass. Six years earlier, she had been told her life expectancy was less than 6 months; overwhelmed by fear and anxiety, she declined further investigation and treatment despite her family’s persuasion.

**Figure 1:**
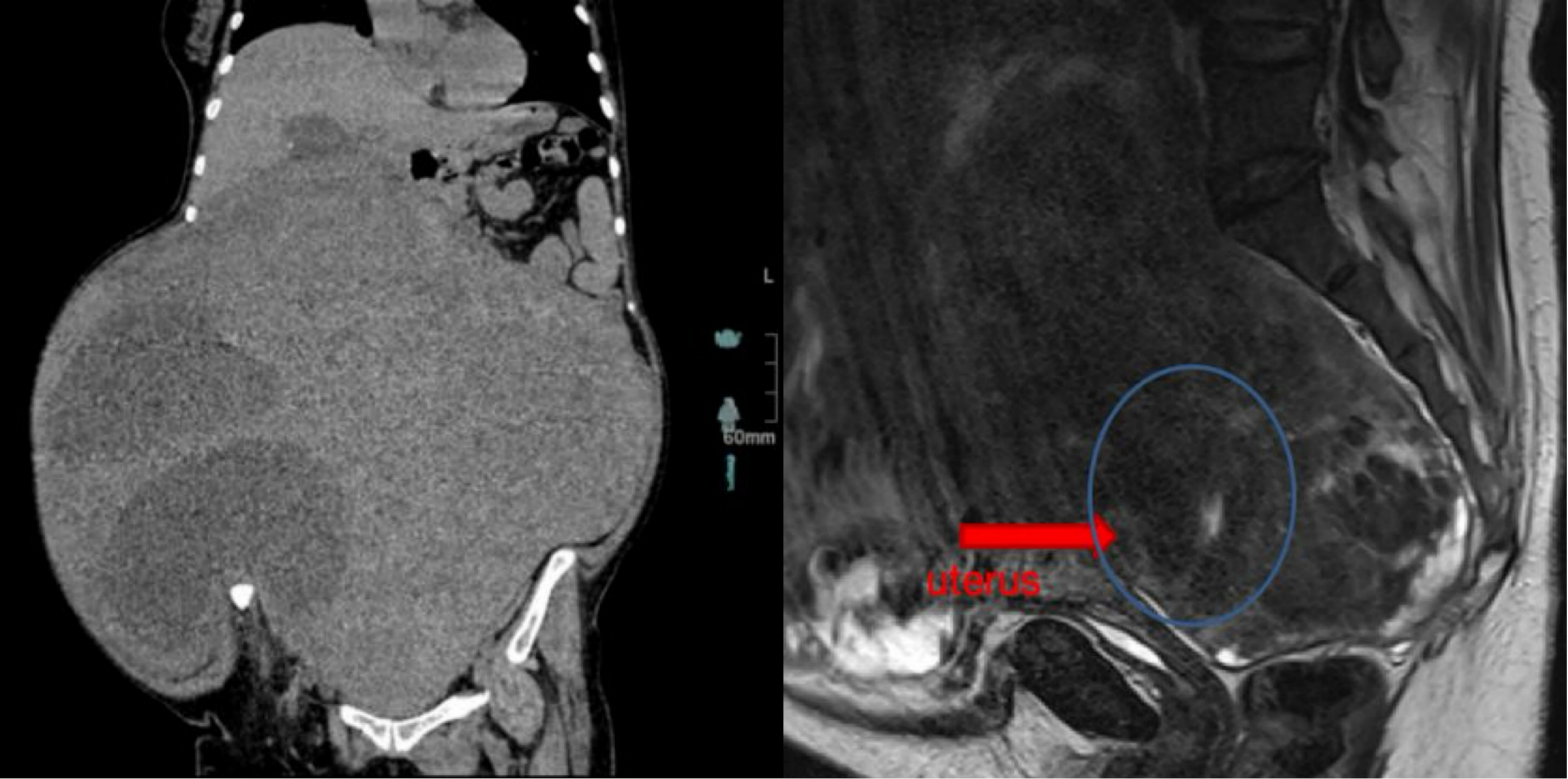
Abdomen and pelvis **(a)** CT. **(b)** MRI.

The patient admits to the gynecology department of our hospital this time due to “recurrent fever for one week, the peak body temperature reached 38.5 °C”. Abdomen and pelvis CT routine scan (**Figure 1a**) indicates that there is a large cystic-solid mass in the pelvic and abdominal cavities, which is suspected to originate from the adnexa. All relevant tumor markers are within normal ranges, including CA125, CA19-9, CEA, AFP, and HE4.Since the onset of the disease, the patient has experienced nausea and oliguria for 1 week. The patient has lost 4 kg in weight over the past week.

To evaluate her conditions, an additional contrast-enhanced MRI (**Figure 1b**) was performed. MRI revealed a large, heterogeneously enhancing pelvi-abdominal mass with mixed signal intensity and internal cystic components, measuring approximately 40×38 cm^2^ in the cross section. The lesion occupied the entire abdominopelvic cavity, and the demarcation from the uterus and adnexa was indistinct.

**Figure 2:**
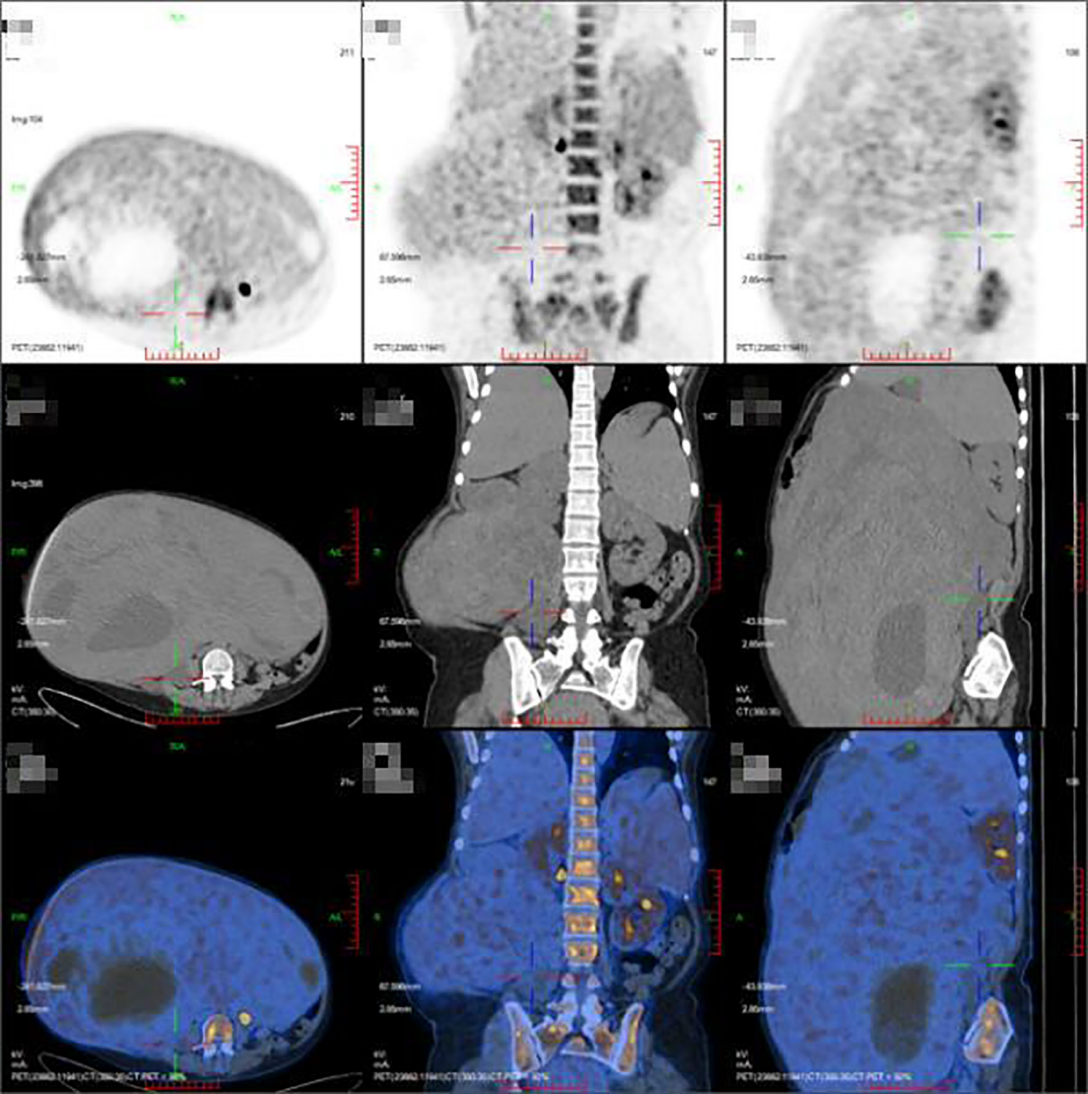
PET-CT.

Subsequently, to distinguish between the benign and malignant characteristics of this mass and perform a thorough and precise assessment, a PET-CT (**Figure 2**) examination was carried out. A large cystic-solid mixed mass was detected in the abdominopelvic cavity. This mass was predominantly solid, with a well-defined border. It extended up to the level of the xiphoid process and grew laterally to the right through the space between the 6th-7th right anterior costal cartilages and the xiphoid process, displacing adjacent tissues. PET-CT showed that its size was approximately 41.2 × 27.8 × 40.6 cm^3^. The density was heterogeneous, and there were multiple focal areas of abnormally intense radioactive accumulation; SUVmax is 4.7. PET-CT suggests a large mixed mass with a mild increase in local metabolism (primarily considered as a benign or low-grade malignant tumor of reproductive system origin), mainly suspected to be a sex cord-stromal tumor or leiomyoma.

The remaining test results indicated that the white blood cell count (WBC) was 17.92×10^9^/L, hemoglobin (HGB) was 100 g/L, the percentage of neutrophils (Neu%) was 91.3%, C-reactive protein (CRP) was 259.95 mg/L, D-dimer was 2.27 μg/mL, estradiol (E2) was 51.98 pg/mL, albumin (ALB) was 32.7 g/L, procalcitonin (PCT) was 0.812 ng/mL, and anti-Müllerian hormone (AMH) was 0.016 ng/mL.

Although the imaging report suggests that the tumor originated from within the abdominal cavity, it is still necessary to differentiate it from a retroperitoneal tumor. A massive retroperitoneal mass has two main diagnostic categories: neoplastic (benign or malignant) and infectious/inflammatory. Malignant causes include retroperitoneal sarcoma (especially liposarcoma and leiomyosarcoma), GIST, lymphoma, and metastases. Benign tumors include schwannoma, paraganglioma, and mature cystic teratoma.

Upon admission, antibiotics (cefoperazone sodium and sulbactam sodium, levornidazole disodium phosphate) were administered to treat infections, heparin sodium was used for thrombus prophylaxis, iron sucrose was given to correct anemia, and albumin was infused to correct hypoalbuminemia. Before the operation, the gynecological team held a multidisciplinary team (MDT) discussion with the cardiology department, urology department, general surgery department, anesthesiology department, radiology department, nutrition and metabolism department, and the nursing team to discuss the perioperative management of the patient, intraoperative respiratory and circulatory support, and various possible crises after the operation. They formulated response strategies and liberation plans one by one.

**Figure 3:**
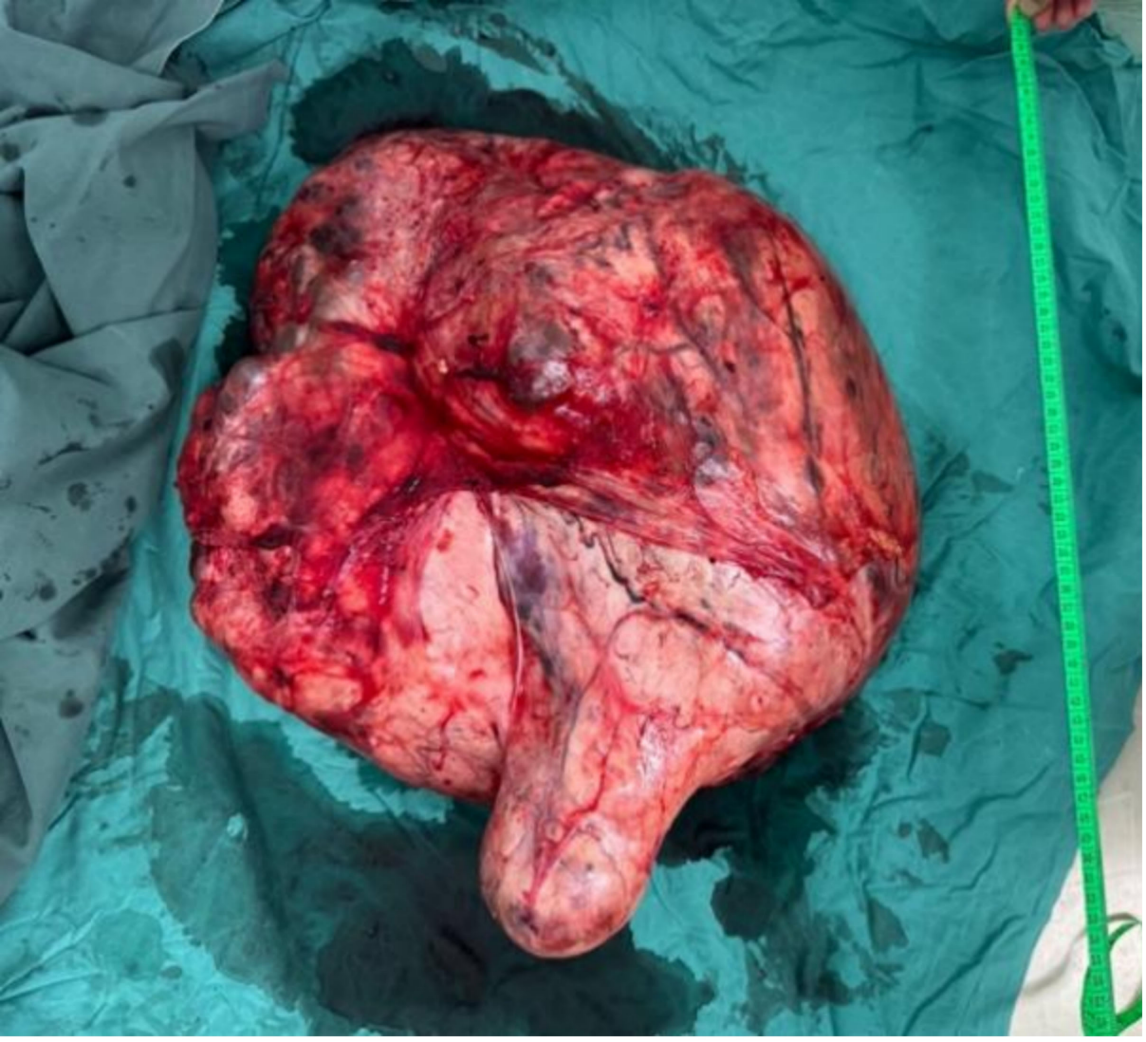
The resected tumor specimen. Size is approximately 48×48×25cm^3^.

Given the patient’s severe infection and compression symptoms and in order to avoid the infection from worsening as a result of the biopsy, after anti-infection and correction cycle treatment, the patient directly underwent surgical treatment. We performed a giant retroperitoneal tumor resection through the abdominal approach, total hysterectomy with bilateral salpingo-oophorectomy, and right fallopian tube stent placement under direct vision. After the anesthesia was completed and the disinfection was done, a 30-cm midline longitudinal incision was made. The abdomen was opened layer by layer to enter the abdominal cavity. Due to the huge size of the tumor, the entire appearance of the tumor and the adjacent pelvic and abdominal organs could not be exposed. The surface of the tumor was covered with a grayish-white tumor capsule and large blood vessels (**Figure 3**). Indeed, it pertains to the retroperitoneum. The uterus was located on the left side of the pelvis. The left fallopian tube was visible. The left ovary was atrophied. The right fallopian tube and round ligament are located in front of the tumor and its capsule, and the right ovarian tissue is not clearly displayed. Using the technique of sharp and blunt dissection, this mass was progressively dissected, elevated, and excised from the retroperitoneal. Considering that the redistribution of volume after the huge mass was lifted could lead to heart failure and pulmonary edema, a warm water bag was used to apply pressure *in-situ* during the slow lifting process. The warm water bag is approximately 15×10×5 cm^3^ in size and weighs about 800 g. It was maintained at a temperature of approximately 38 °C and applied over the inferior vena cava and abdominal aorta for a duration of about 25 min. Concurrently with the compression, hemodynamic monitoring was performed: heart rate, blood pressure, respiratory rate, and oxygen saturation were recorded at 10-min intervals, and central venous pressure was monitored simultaneously. Subsequently, the retroperitoneal organs and vessels were exposed. The abdominal aorta, inferior vena cava, common iliac arteries on both sides, and the right kidney were all displaced toward the midline of the abdomen.

The resected specimen measured 48×48×25 cm³ and weighed 40 kg. Cross section revealed predominantly muscular tissue with cystic components and grayish-yellow necrotic areas with a fetid odor.

During the operation, eight units of leukocyte-reduced red blood cells and 500 ml of fresh frozen plasma were transfused. Postoperatively, the blood pressure was 90/60 mmHg, the heart rate was 105 beats per minute, and the blood oxygen saturation was 100%. The patient was transferred to the Medical Intensive Care Unit (MICU) for close observation.

The patient was hospitalized in the MICU for 3 days. During this period, anti-shock treatment, correction of electrolyte imbalance, correction of hypoproteinemia, blood transfusion to correct anemia, anti-infection, and symptomatic treatment were provided. Her postoperative Clavien-Dindo grading was grade II. The patient’s condition gradually improved and was transferred back to the gynecology ward for further treatment.

**Figure 4:**
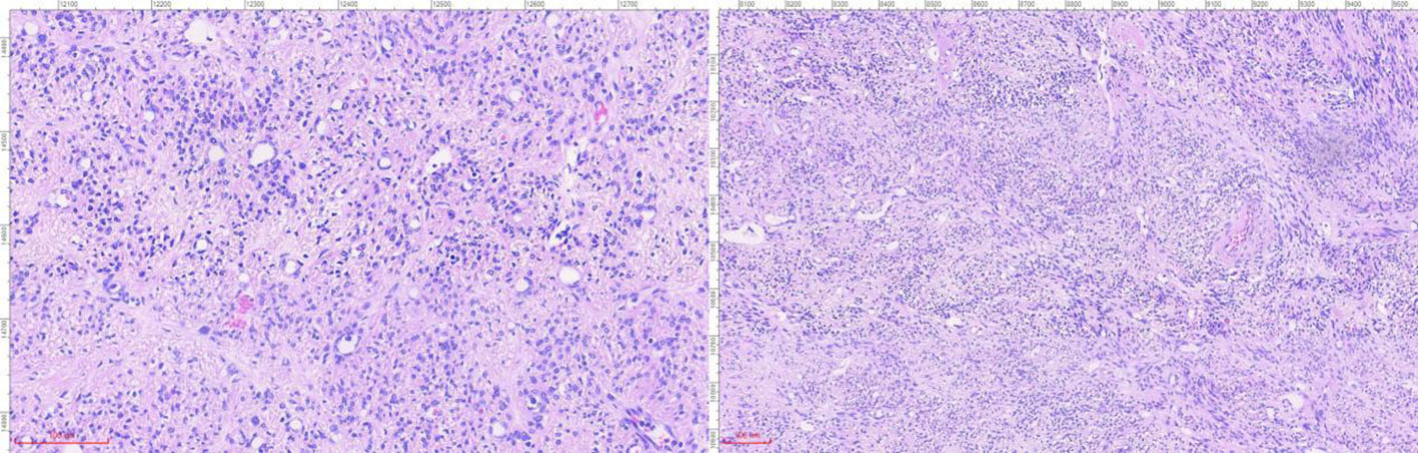
Histopathology figures with H&E staining at low and high magnifications.

Postoperative pathology (**Figure 4**) indicates (1) spindle cell neoplasm with hyaline degeneration and infarction, consistent with leiomyoma, without evidence of malignancy; (2) multiple leiomyomas of the uterus; (3) postmenopausal endometrium; (4) chronic cervicitis; and (5) no abnormal lesions in bilateral fallopian tubes and left ovary.

Psychological intervention was provided by a licensed clinical psychologist and integrated into the patient’s perioperative care pathway. Preoperatively, after the initial Hamilton Anxiety Scale (HAMA) assessment (score 21), the patient received two 1-h sessions of supportive psychotherapy combined with cognitive–behavioral techniques, focusing on illness perception, fear of recurrence, and sleep disturbance. During the 1-week postoperative hospitalization, three additional bedside sessions were conducted to address body image concerns, pain-related anxiety, and adherence to medication and follow-up. At discharge, the patient was referred for continued outpatient biweekly psychotherapy for 8 weeks and was scheduled for repeated HAMA assessment at postoperative weeks 2, 4, and 12, with the first follow-up already showing a score of 9 (≥29 points: severe anxiety; ≥21 points: obvious anxiety; ≥14 points: definitely have anxiety; ≥7 points: possibly have anxiety; <7: no anxiety).

On the day of discharge, the WBC count was 7.01×10^9^/L, the HGB level was 104 g/L, the D-dimer level was 4.84 µg/mL, and the albumin level was 39.3g/L. The VTE score is 8 points. Given that the D-dimer level of the patient was above the abnormal value upon admission and remained elevated postoperatively, and despite anticoagulation and thrombosis prevention treatment, the D-dimer level remained abnormal. Regular color Doppler ultrasound examinations of the lower extremities have shown no abnormalities. It is considered to be related to the physiological coagulation activation after surgery and perioperative stress. The patient was instructed to wear thromboembolic compression stockings after discharge, take rivaroxaban tablets orally, and have regular blood routine and liver and kidney function tests. The patient has recovered well; she visited the urology department 2 months after the operation and underwent a ureteral stent removal. After the stent was removed, hydronephrosis occurred. Currently, the patient is under close follow-up and treatment in the urology department.

## Discussion

This case strongly demonstrates that the huge volume of retroperitoneal mass is not necessarily related to malignancy. Although the tumor in this case presented to be extremely invasive on imaging and caused severe physiological compression and complications, its pathological nature was still benign. Given the unprecedented tumor size, the theoretical possibility of an atypical presentation of hereditary leiomyomatosis and renal cell carcinoma (HLRCC) syndrome cannot be entirely excluded.

This case has two notable strengths. First, an innovative warm water bag compression technique provided a simple, low-cost, and titratable method for preventing hemodynamic collapse during tumor removal, offering a practical alternative to abdominal binding or staged resection. Second, the structured integration of psychological intervention with quantitative HAMA assessment exemplifies a holistic perioperative model rarely documented in surgical case reports. HAMA is used to quantify perioperative anxiety severity and to monitor response to psychological interventions in surgical patients (8).

To date, the intraoperative application of warm-water bag compression remains scarcely documented in the surgical literature. For major abdominal surgery, goal-directed hemodynamic monitoring employing calibrated pulse contour analysis or transpulmonary thermodilution is recommended to evaluate cardiac output, fluid responsiveness, and dynamic preload indicators. Dynamic parameters and individualized perfusion targets, instead of static thresholds, are utilized to direct fluid and vasoactive therapy and minimize perioperative complications (9).

Iatrogenic ureteral injuries occur most frequently in gynecological surgery. Preoperative ureteral stent placement or intravenous injection of ICG during the operation can reduce the injury to the ureter during the operation. Despite standard intraoperative precautions including visual inspection, the massively distorted anatomy led to an inadvertent transection of the displaced ureter. If ureteral injury is found during the operation, ureteroureterostomy can be performed and a double-J ureteral stent can be placed (10). Long-term follow-up by a urologist is required after the operation. Ureteral patency should be assessed 4–6 weeks after stent removal, followed by annual urological surveillance.

This case underscores the importance of integrating empathetic psychological support into perioperative care to build trust and improve outcomes.

## Patient perspective

The patient described her decade-long journey: “I felt the lump ten years ago but was too afraid to seek help. When a doctor told me six years ago I might only have six months to live, I gave up completely. By the time I came to this hospital with fever and inability to urinate, I had lost all hope. This time, however, the team did not abandon me. The psychologist helped me understand my fear. Waking up after surgery and learning the 30 kg tumor was benign, I wept with relief. The care I received treated not just my disease but restored my will to live.”

## Data Availability

The original contributions presented in the study are included in the article/supplementary material. Further inquiries can be directed to the corresponding author.
